# GOBO projection for 3D measurements at highest frame rates: a performance analysis

**DOI:** 10.1038/s41377-018-0072-3

**Published:** 2018-10-03

**Authors:** Stefan Heist, Patrick Dietrich, Martin Landmann, Peter Kühmstedt, Gunther Notni, Andreas Tünnermann

**Affiliations:** 1 Institute of Applied Physics, Abbe Center of Photonics, Friedrich Schiller University, 07745 Jena, Germany; 2 Fraunhofer Institute for Applied Optics and Precision Engineering IOF, 07745 Jena, Germany; 3 Department of Mechanical Engineering, Ilmenau University of Technology, 98693 Ilmenau, Germany

## Abstract

Aperiodic sinusoidal patterns that are cast by a GOBO (GOes Before Optics) projector are a powerful tool for optically measuring the surface topography of moving or deforming objects with very high speed and accuracy. We optimised the first experimental setup that we were able to measure inflating car airbags at frame rates of more than 50 kHz while achieving a 3D point standard deviation of ~500 µm. Here, we theoretically investigate the method of GOBO projection of aperiodic sinusoidal fringes. In a simulation-based performance analysis, we examine the parameters that influence the accuracy of the measurement result and identify an optimal pattern design that yields the highest measurement accuracy. We compare the results with those that were obtained via GOBO projection of phase-shifted sinusoidal fringes. Finally, we experimentally verify the theoretical findings. We show that the proposed technique has several advantages over conventional fringe projection techniques, as the easy-to-build and cost-effective GOBO projector can provide a high radiant flux, allows high frame rates, and can be used over a wide spectral range.

## Three-dimensional imaging: Revolving light patterns hold the key

High-speed projection of light patterns is enabling scientists in Germany to image moving three-dimensional objects. Capturing the minute surface details of moving or deforming objects, such as car airbags being deployed, could hold great value for industry. Stefan Heist at Friedrich Schiller University and Fraunhofer IOF in Jena, Germany, and co-workers have used a GOBO (GOes Before Optics) projector together with two cameras to create a sensor for measuring 3D shapes. The GOBO projects a continually changing pattern onto an object of interest, and the pattern is picked up by the cameras. The team calculated the optimal design of the aperiodic light patterns to generate a dense 3D cloud of data points. Their system accurately captured and reconstructed three-dimensional dynamic processes such as a ball being kicked, at a resolution of one megapixel.

## Introduction

Measuring the three-dimensional (3D) topography of macroscopic objects by using structured light requires

(i) the (sequential) projection of *N* ≥ 1 pattern(s) onto the object and
(ii) the simultaneous recording of the pattern(s) that are modulated by the object topography.

Years of research and development have shown that the accuracy that can be achieved by such pattern projection systems depends directly on the number *N* of projected patterns^1–3^. Along with the increased demands on measurement accuracy, in recent years, requirements on measurement speed have also risen, which necessitate high-speed pattern projection and recording and fast computation and evaluation. In particular, dynamically moving or deforming objects are to be measured.

In general, well-known algorithms for determining 3D object coordinates by evaluating projected patterns are based on detecting two-dimensional (2D) point correspondences between two cameras or between one camera and the projector^4–7^. Corresponding points are defined as 2D sensor points that are images of the same 3D object point. When using a series of *N* patterns, triangulation algorithms require temporal consistency of these point correspondences during the period *NT* = *N*/*f*, where *f* = *T*^−1^ is the projection and recording frame rate. In dynamic measurement situations, i.e., if the measurement object and the sensor system move relative to each other, this rigid assignment will not be satisfied.

To re-establish a quasi-static measurement scenario, two approaches are possible: first, methodological modifications could be made, e.g., reducing the number of patterns *N* and, therefore, the period *NT*; projecting alternative patterns; or compensating for the relative movement. In particular, single-shot techniques (*N* = 1), such as Fourier transform profilometry^8,9^, multi-line triangulation^10^, and wave grid-based active stereo^11^, are popular and efficient ways to minimise the measurement time. Second, the projector and camera frame rate could be significantly increased. The minimum of the two frame rates limits the measurement speed. In contrast to cameras with high sensitivity, frame rate, and resolution (several kHz at megapixel resolution), which have been commercially available for some time, conventional projectors are limited in terms of speed (especially in 8-bit greyscale mode), radiant flux, and applicable light spectral range^12–14^. Thus, the focus is on improving traditional projection systems, along with making potentially necessary changes in the design of the projected patterns.

In addition to the well-known and extensively studied phase-shifting fringe projection and phase value calculation^15–17^, 3D object coordinates can be determined by evaluating a pattern sequence via temporal correlation^18,19^. At each image point (*x*^(1)^,*y*^(1)^) in camera 1, a temporal grey value sequence $I_1^{\left( 1 \right)}, \ldots ,I_N^{\left( 1 \right)}$ is measured and correlated with the grey value stack $I_1^{\left( 2 \right)}, \ldots ,I_N^{\left( 2 \right)}$ of each pixel (*x*^(2)^,*y*^(2)^) in camera 2 according to the normalised cross-correlation:

1 $$\rho = \frac{{\mathop {\sum }\nolimits_{i = 1}^N \left[ {I_i^{\left( 1 \right)} - \overline {I^{\left( 1 \right)}} } \right]\left[ {I_i^{\left( 2 \right)} - \overline {I^{\left( 2 \right)}} } \right]}}{{\sqrt {\mathop {\sum }\nolimits_{i = 1}^N \left[ {I_i^{\left( 1 \right)} - \overline {I^{\left( 1 \right)}} } \right]^2} \sqrt {\mathop {\sum }\nolimits_{i = 1}^N \left[ {I_i^{\left( 2 \right)} - \overline {I^{\left( 2 \right)}} } \right]^2} }}$$

where $\overline {I^{\left( j \right)}}$ is the arithmetic mean of the grey value sequence in camera *j*. Corresponding points are obtained by maximising the correlation coefficient *ρ*. If the system parameters are calibrated, 3D object coordinates can be calculated via triangulation of corresponding points^20,21^.

In contrast to phase-shifting methods, determining corresponding points via normalised cross-correlation does not require any knowledge of the pattern design or the variation between successive patterns. To be suitable for (dynamic) 3D measurements, the only prerequisites are a significant temporal variation of the intensity distribution and spatial frequencies that match the other system parameters. The camera resolution, magnitude of the pattern variation, and spatial frequencies of the patterns should be fine-tuned to minimise disturbing effects and obtain the optimum reconstruction accuracy.

Aperiodic sinusoidal fringes are a special type of temporal pattern coding^22^. In contrast to two-dimensionally varying patterns, such as speckle patterns^23,24^, aperiodic sinusoidal fringes vary solely in one dimension:

2 $$I_i^{\rm{{proj}}}\left( {x,y} \right) = a_i\left( x \right) + b_i\left( x \right)\sin \left[ {c_i\left( x \right)x + d_i\left( x \right)} \right]$$

with spatially and temporally varying offset *a*_*i*_(*x*), amplitude *b*_*i*_(*x*), period length 2*π*/*c*_*i*_(*x*), and phase shift *d*_*i*_(*x*). The loss of coding information in one dimension is compensated for by making use of the sensor geometry. Based on the extrinsic and intrinsic camera parameters, the search space for point correspondences can be reduced to so-called epipolar lines^20,21^. Then, an intensity variation along these lines is sufficient, i.e., the aperiodic sinusoidal fringes should be approximately perpendicular to the epipolar lines.

One novel approach to high-speed pattern projection is the GOBO projection of aperiodic sinusoidal fringes^25^. In general, GOBO projectors consist of a light source, a light collector, a slide (GOBO = GOes Before Optics), and imaging optics. Changing the projected patterns can be realised by moving the GOBO, e.g., by rotating a GOBO wheel. To project aperiodic sinusoidal fringes, the GOBO wheel is equipped with aperiodic binary fringes, the projected image is slightly defocused, and the GOBO wheel is revolving during the camera exposure time. In this way, the wheel can be rotated continuously instead of in start/stop operation. Furthermore, the projector does not need to be synchronised with the cameras.

When using an appropriate light source, a GOBO projector can provide a radiant flux of several 100 W, thereby allowing for extremely low camera exposure times in the range of a few microseconds. With a 3D sensor that comprises a GOBO projector and two high-speed cameras, we were able to three-dimensionally capture highly dynamic processes, such as a soccer ball kick. The system enabled us to reconstruct 1300 independent point clouds per second at a resolution of 1 megapixel^25^. Higher frame rates of more than 50 kHz can be achieved when reducing the camera resolution and adjusting the rotational speed of the GOBO wheel accordingly.

After demonstrating the suitability of a GOBO projector for high-speed 3D measurements via mainly qualitative studies, quantitative investigations are necessary. In this paper, we theoretically study the dependency of the 3D reconstruction quality on various parameters of the GOBO projection-based system, e.g., the GOBO wheel’s rotational speed and the cameras’ exposure time.

## Results

The quality of a 3D point cloud can be characterised by two crucial indicators: accuracy and completeness. When measuring an object with a GOBO projection-based sensor, occlusions might restrict the surface area that is covered by both the projector and the cameras, which limits the maximum number of points that can be reconstructed. The completeness *p* of a 3D point cloud specifies how many of these points have been correctly determined. The accuracy can be described by the standard deviation *σ*_3D_ of non-outlier points from the known surface. Naturally, the completeness should be as high as possible, i.e., *p* = 100%, and the standard deviation should be as low as possible. Therefore, the parameters of a GOBO projection-based 3D sensor, such as the distance between the cameras, the working distance, the number of strips and slits in the GOBO wheel, and its rotational speed, must be carefully designed to match one another.

**Fig. 1:**
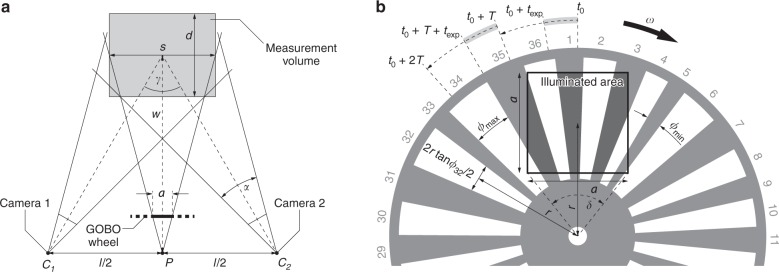
**Schematic illustration of a GOBO projection-based 3D sensor.** Definitions of some of the variables that are listed in Table 1 by means of **a** a top view of the camera-projector-camera arrangement and **b** an exemplary GOBO wheel with aperiodic binary fringes

Table 1 summarises the parameters of a GOBO projection-based 3D sensor, which affect the accuracy and the completeness of the 3D reconstruction. Some of these variables are related to each other, e.g., the number of strips and slits in the GOBO wheel and the average angle that is covered by one strip or slit. The third column lists values that are realised in the current setups. To facilitate understanding of the parameters, Fig. 1 illustrates some of the variables that are shown in Table 1. Figure 1a shows a schematic top view of the camera-projector-camera arrangement. The projection centre, which is denoted by *P*, should be midway between the two camera centres, which are denoted as *C*_1_ and *C*_2_. The principal rays of the cameras and the projector intersect at the centre of the cuboid measurement volume. Figure 1b shows an exemplary GOBO wheel with 36 aperiodic binary fringes. Only a square area of size *a* × *a* is illuminated and imaged into the measurement volume. According to their frame rate *f* = *T*^−1^, both cameras start acquiring an image at any time *t*_0_ + *kT*, $k \in {\Bbb N}$. Throughout the exposure time *t*_exp_, the GOBO wheel is continuously rotating through an angle of *t*_exp_*ω*.

**Table 1:** Parameters of a GOBO projection-based 3D sensor that affect the accuracy and completeness of the 3D result

Parameter	Description	Typ. value	Sim. value
*N *≥ 3, $N \in {\Bbb N}$	Number of patterns per sequence	8…12	10
*n*_tot_ = 2*k*, $k \in {\Bbb N}$	Number of strips + number of slits in the GOBO wheel	1200…2000	250…5000
$\phi _{{\rm{avg}}} = \frac{{360^\circ }}{{n_{{\rm{tot}}}}}$	Average angle that is covered by one strip or slit	0.18°…0.3°	0.1°…1.5°
$c = \frac{{\phi _{{\rm{max}}}}}{{\phi _{{\rm{min}}}}} \ge 1$	Ratio of the maximum and minimum angles that are covered by one strip or slit	2	1…10
*P*(*φ*)	Probability distribution of the strip or slit angle *φ*	Uniform dist.	Uniform dist.
*r* > 0	Distance of illuminated square’s centre from the GOBO wheel’s centre	20…200 mm	25 mm
*a* > 0	Width (= height) of the illuminated square on the GOBO wheel	10…100 mm	10 mm
$\delta = 2\,\arctan \frac{{a/2}}{{r - a/2}}$	Maximum angle that is covered by the illuminated square	15°…30°	28.1°
0 < *n* < *n*_tot_	Number of illuminated strips + number of illuminated slits	50…150	20…400
*σ*_blur_ ≥ 0	Projector defocusing, which is approximated by Gaussian blur with std. dev. *σ*_blur_	$0.2r\tan \frac{{\phi _{{\rm{avg}}}}}{2}$	0…50 µm
*s* > 0	Width (= height) of the measurement volume	0.2…2 m	0.3 m
*d* > 0	Depth of the measurement volume	0.2…1 m	0.3 m
$v = \frac{s}{d} > 0$	Ratio of the width *s* and depth *d* of the measurement volume	0.5…2	1
$M = \frac{s}{a} > 0$	Magnification of the GOBO pattern	10…50	30
*w* > 0	Working distance (projection centre to measurement volume’s centre)	0.5…4 m	1 m
$\omega = \frac{\varphi }{T} > 0$	Rotational speed of the GOBO wheel, which is given by the covered angle *ϕ* between two images from cameras at frame rate *f* = *T*^−1^	$0.5\frac{{\phi _{{\rm{avg}}}}}{T} \ldots \frac{{\phi _{{\rm{avg}}}}}{T}$	$\frac{{0.001^\circ }}{T} \ldots \frac{{2.5^\circ }}{T}$
$0 < e = \frac{{t_{{\rm{exp}}}}}{T} \le 1$	Ratio of the camera exposure time *t*_exp_ and period *T* = *f*^−1^	0.6…0.95	0.5…1
*l* > 0	Distance between the two camera centres	0.1…1 m	0.2 m
$\gamma = 2\,\arctan \frac{{l/2}}{w}$	Triangulation angle between the optical axes of the cameras	10°…30°	11.42°
*α*	Horizontal (= vertical) field of view of the cameras	15°…40°	16.2°
*A*	Camera resolution	0.25…4 Mpx	1 Mpx
*b*	Camera bit depth	8 bit, 12 bit	8 bit
$\sigma _e^2$, $\sigma _d^2$, $\sigma _q^2$	Camera noise (shot noise, dark noise, analogue-to-digital conversion)^28^	–	0

The grey highlighted cells indicate parameters that we have varied in a simulation. (Parameters *n*_tot_ and *Φ*_avg_ are implicitly also varied, but depend on *n*.)

### Aperiodic sinusoidal patterns

**Fig. 2:**
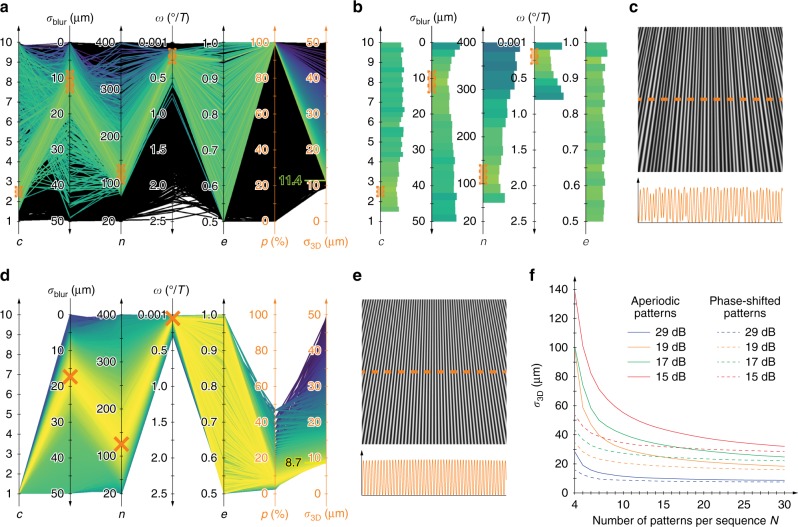
3D point cloud completeness *p* and standard deviation *σ*_3D_ when using varying GOBO wheel parameters to project aperiodic sinusoidal patterns (upper row) and phase-shifted sine-like patterns (lower row). **a**, **d** *p* and *σ*_3D_ as functions of randomly varied parameters *c*, *σ*_blur_, *n*, *ω*, and *e*. **b** The minimum standard deviation *σ*_3D_, which is shown for each of the five parameters. **c**, **e** A camera image of the projection of the optimal GOBO pattern onto a plane. **f** The 3D point standard deviation *σ*_3D_ as a function of the number of aperiodic sinusoidal patterns (solid lines) and phase-shifted sine-like patterns (dashed lines) that are used for reconstruction

Using the simulation framework that is presented in the Methods section, we generated several thousand GOBO wheels with random parameters *c* (the ratio of the maximum and minimum strip or slit widths), *σ*_blur_ (the degree of defocusing of the GOBO wheel), and *n* (the number of illuminated strips + the number of illuminated slits). Then, we rendered camera images according to random parameters *ω* (the rotational speed of the GOBO wheel) and *e* (the ratio of camera exposure time *t*_exp_ and period *T*), added noise that corresponded to four signal-to-noise ratios (SNRs), and calculated 3D point clouds. For the exclusive presence of quantisation noise (SNR ≈ 29 dB), Fig. 2a shows the results that have standard deviations of *σ*_3D_ = 50 µm or lower, which are represented in parallel coordinates. Parameter sets that lead to a completeness of *p* = 100% are coloured according to the standard deviation. Parameter sets that result in a completeness of *p* < 100% are shown in black (background). For some of the parameters, the green curves, which correspond to small standard deviations, are concentrated around certain values (see the orange rectangles).

For each of five parameters, namely, *c*, *σ*_blur_, *n*, *ω*, and *e*, Fig. 2b shows the minimum standard deviation *σ*_3D_ of the point clouds with a completeness *p* = 100% that have been reconstructed during the simulation. In this representation, the optimal ranges become apparent, which are indicated by orange rectangles. They enable the derivation of general guidelines for designing an optimum GOBO wheel for a specified sensor:

- Based on the optimum range of the number *n* of illuminated strips and slits, the average fringe pitch should be approximately 22 px in the camera images. A larger fringe width results in a reduced measurement accuracy, while a smaller fringe width reduces the uniqueness of the sequence, thereby leading to a lower completeness.
- The exposure time proportion, which is expressed as $e = \frac{{t_{{\rm{exp}}}}}{T}$, does not exhibit a distinct minimum, i.e., there are accurate data sets for each value of *e*. For that reason, and since an exposure time proportion that is as large as possible is desired for high-speed measurements, it can be fixed to a reasonable value of *e* = 0.95, which takes the short time of 0.05*T* for data readout into account.
- The rotational speed *ω* of the GOBO wheel should be such that the pattern is rotated by nearly half the average fringe pitch between each image acquisition. A higher rotational speed would ensure that very different areas of the GOBO wheel are illuminated successively so that the temporal intensity values are independent of each other. However, this would lead to substantial blurring of the fringes during the exposure time and, thus, to an undesired low grey value modulation.
- The defocusing of the imaging lens should be adjusted such that the rotating pattern neither contains intensity plateaus nor has a poor modulation. If the projection is too sharp, the integration of the rotating pattern over the exposure time results in triangular or trapezoidal patterns instead of aperiodic sinusoidal patterns. If the projection is too blurry, the grey values of adjacent camera pixels do not differ substantially.
- The ratio *c* of the maximum and minimum angles that are covered by one strip or slit should be between 2 and 2.5. The closer the ratio is to 1, the more periodic and, therefore, ambiguous the patterns become, such that completeness of 100% cannot be achieved. The larger the ratio, the more inhomogeneously the fringes are blurred. Either narrow fringes with very low modulation or broad fringes with unwanted intensity plateaus are obtained.

The randomness of the fringe generation can lead to different standard deviations *σ*_3D_ and completeness values *p* for the same parameter set, which made it impossible for us to carry out any direct optimisation. Even if, e.g., we used the average or median of many results for each parameter set, optimisation algorithms became trapped in different local extrema each time, despite unchanged initial values. Therefore, within the restricted parameter ranges (see the orange rectangles in Fig. 2a, b; *e* = 0.95), we have generated several thousand additional random GOBO patterns and picked the one with *p* = 100% completeness that yielded the lowest standard deviation *σ*_3D_. The optimal result was the one that is shown in Table 2 for SNR ≈ 29 dB with a completeness of *p* = 100% and a standard deviation of *σ*_3D_ ≈ 11.4 µm (which is equal to a relative standard deviation $\sigma _{{\rm{3D}}}^{{\rm{rel}}} = \sigma _{{\rm{3D}}}/\sqrt {2s^2 + d^2} \approx 2.2 \times 10^{ - 5}$ with respect to the measurement volume diagonal). Figure 2c shows one of the corresponding camera images and a profile along the central line. Based on this optimal projected pattern, the full GOBO wheel can be constructed via repetition of this section so that the results remain approximately unchanged for all rotation angles of the GOBO wheel.

**Table 2:** Exemplary results of 3D point cloud completeness *p* and standard deviation *σ*_3D_ for parameters *c*, *σ*_blur_, *n*, *ω*, and *e*

	Aperiodic sinusoidal patterns				Phase-shifted patterns			
Parameter	29 dB	19 dB	17 dB	15 dB	29 dB	19 dB	17 dB	15 dB
*c*	2.2	2.5	2.7	3.0	1.0			
*σ*_blur_	12 µm	6 µm	4 µm	3 µm	17 µm	8 µm	6 µm	5 µm
*n*	120	230	280	330	130	260	332	400
*ω*	0.21°/*T*	0.11°/*T*	0.09°/*T*	0.08°/*T*	0.04°/*T*	0.02°/*T*	0.01°/*T*	0.01°/*T*
*e*	0.95				0.95			
*p*	100%	100%	100%	100%	7.1%	3.6%	2.8%	2.3%
*σ*_3D_	11.4 µm	31.2 µm	41.7 µm	55.1 µm	8.7 µm	20.5 µm	27.1 µm	34.0 µm
$\sigma _{{\rm{3D}}}^{{\rm{rel}}}$	0.022‰	0.060‰	0.080‰	0.106‰	0.017‰	0.039‰	0.052‰	0.065‰

The randomly obtained optimum agrees very well with the results of previous theoretical and experimental investigations on aperiodic sinusoidal fringes^26,27^. In these investigations, we used the same sensor setup and obtained an optimum of 90 fringes within the measurement field. For GOBO-projected aperiodic sinusoidal patterns, the number of projected fringes inherently varies vertically due to the wheel layout. Therefore, the number of illuminated strips and slits, namely, *n* = 120, that was obtained for the parameter set in Table 2 is only realised in the upper part of the camera image. In the centre, the number of fringes is *n *≈ 90 (see Fig. 2c), which corresponds very well to the values that were obtained in previous examinations.

Up to this point, we have taken into account spatial integration over the camera pixels and 8-bit quantisation in our simulation. However, cameras of real 3D sensors will exhibit two additional types of noise, which negatively affect the accuracy that can be achieved with the optimised patterns: shot noise and dark noise. We simulated the signal-dependent shot noise *n*_*e*_ (Poisson distribution with expected value *μ*_*e*_ and standard deviation $\sigma _e = \sqrt {\mu _e}$) and the signal-independent dark noise *n*_*d*_ (normal distribution with standard deviation *σ*_*d*_) according to EMVA standard 1288^28^ as described in the Methods section. We considered three levels of noise that correspond to signal-to-noise ratios of SNR ≈ 19 dB, 17 dB, and 15 dB. The following conclusions are drawn from the simulation results.

- The lower the SNR, the lower the 3D point accuracy and the fewer pattern sets result in 100% point cloud completeness. High noise leads to a high standard deviation *σ*_3D_. However, noisy data can also cause the temporal grey value sequences of non-corresponding points to have a higher correlation coefficient than the actual homologous points. For this reason, for an SNR of 15 dB, 10 patterns are insufficient for achieving 100% completeness.
- The lower the SNR, the higher the number of fringes that are necessary to obtain high accuracy, which is caused by the counteraction of two effects: For broad fringes, the integrated intensity over the finite area of a pixel approximates very well the projected intensity in the centre of the pixel. For narrow fringes, the grey values of adjacent pixels differ significantly, thereby making subpixel interpolation more reliable. The higher the noise, the more essential it is to have a large difference between adjacent grey values.

Table 2 shows the optimum GOBO parameters that we obtained for SNR ≈ 19 dB, 17 dB, and 15 dB. Although the span of the standard deviation and completeness increases with decreasing signal-to-noise ratio, it is still possible to generate patterns that yield 100% completeness. For SNR ≈ 15 dB, the 3D point standard deviation is ~4.8 times higher than for 29 dB.

### Phase-shifted sine-like patterns

According to the discussed studies on aperiodic sinusoidal fringes^26,27^, phase-shifted sinusoidal fringes are the limiting case of aperiodic sinusoidal fringes in terms of accuracy. Therefore, it is reasonable to investigate whether this is also the case with GOBO projection because the GOBO projector can be used to project phase-shifted sine-like patterns when synchronised with the cameras^29,30^. In the presented simulation framework (see Methods section), this can be carried out by setting *c* = 1 (the ratio of the maximum and minimum fringe widths) and ω = 2*ϕ*_avg_/*NT* (the rotational speed of the GOBO wheel). Random generation of the remaining parameters, namely, *n*, *σ*_blur_, and *e*, leads to the diagram that is shown in Fig. 2d for SNR ≈ 29 dB.

The comparatively poor completeness of the reconstructed point clouds is noticeable. Due to the periodicity of the sinusoidal patterns, the temporal grey value sequences are ambiguous and applying Eq. 1 yields many false correspondences, as the number of maxima of the correlation coefficient equals the number of fringe pairs within the disparity search range of ≈ ±110 px. These ambiguities must be eliminated via phase unwrapping^31,32^. However, conventional methods that are based on the evaluation of the phase difference between adjacent pixels fail on objects with sharp edges or large depth. Therefore, a variety of techniques have been developed for localising corresponding fringe periods.

The most robust phase unwrapping method is the projection of a sequence of Gray code patterns^33–35^, which leads to a significant increase in the total length of the pattern sequence *N*. Moreover, it is not feasible to project Gray code patterns using the GOBO projection principle. The projection of phase-shifted sinusoidal fringes with different period lengths^36–41^ is also impracticable because this would require divergent rotational speeds of the GOBO wheel. Instead, an additional pattern must be embedded in the sinusoidal pattern, e.g., a one-dimensional binary De Bruijn sequence^42^ or a band-limited 1/*f* noise pattern^43,44^, which can be achieved, for example, by placing an additional (static) slide in front of the GOBO wheel^29,30^.

An embedded pattern reduces the signal-to-noise ratio of the phase values and, thus, the measurement accuracy. In addition, such an approach requires a spatial correlation that acts as a spatial frequency low-pass filter so that high-frequency components of the object surface are not taken into account. Therefore, in all studies of GOBO-projected phase-shifted sinusoidal patterns, we have considered only pure sinusoidal patterns. In the general case of an arbitrarily complex measurement object, these patterns lead to very low completeness. However, they yield high accuracy, as shown by the large number of yellow curves, which correspond to small standard deviations *σ*_3D_, in Fig. 2d. The optimum values of the parameters *σ*_blur_, *n*, and *ω* are indicated by orange crosses. The exposure ratio *e* can again be chosen almost arbitrarily; it is set to 0.95.

In contrast to aperiodic sinusoidal patterns, the remaining independent parameters, namely, *σ*_blur_ and *n*, uniquely specify a phase-shifted sine-like pattern. Therefore, they can be easily optimised, e.g., by the downhill simplex method^45^. The results are listed in Table 2. As expected, phase-shifted sine-like patterns yield the lowest 3D point standard deviation. The achievable standard deviation is between 1.3 and 1.6 times smaller than with aperiodic sinusoidal patterns. However, if there are no special constraints on the measurement object, the periodic pattern cannot be used due to its ambiguities, which lead to poor completeness.

Figure 2e shows the optimum sine-like pattern for SNR ≈ 29 dB. The determined number of fringes is consistent with the findings of previous studies^26,27^, in which an optimal number of 100 fringes was obtained. In the centre of the optimised GOBO-projected phase-shifted pattern, 102 fringes are projected, which demonstrates the high degree of agreement between the investigations.

Naturally, the achievable 3D accuracy depends directly on the number of patterns that are used for the reconstruction. Figure 2f shows this dependence for both phase-shifted and aperiodic sinusoidal patterns. For example, with 10 aperiodic sinusoidal patterns, the same standard deviation is obtained as with 5–6 phase-shifted sinusoidal patterns, which is in line with the results of previous investigations of the measurement technique^26,27^. However, aperiodic sinusoidal patterns yield a significantly higher completeness of *p* = 100%, thereby making resolving ambiguities by projection of additional patterns unnecessary. Therefore, GOBO projection of aperiodic sinusoidal fringes is an excellent alternative to the established phase-shifting technique. In addition, it offers the potential for an extremely fast and bright projection compared to conventional projectors and it can easily be used in a wide spectral range^25^.

### Experimental evaluation

**Fig. 3:**
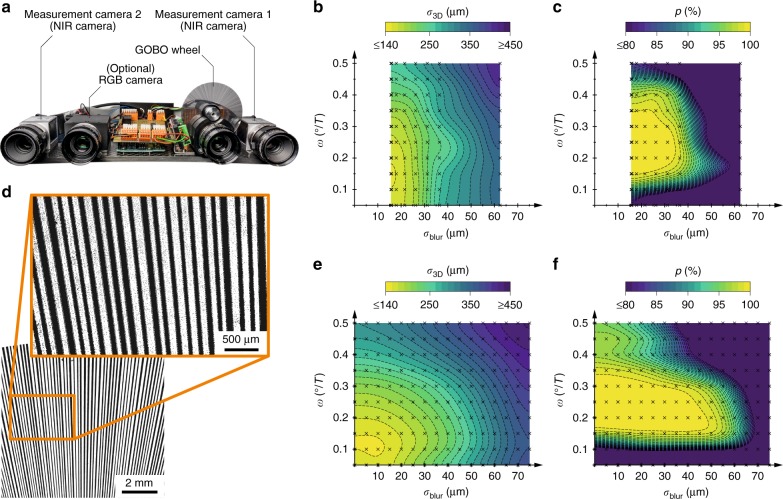
Experimental investigation. **a** A photograph of the sensor that is used. **b**, **c** The measured and **e**, **f** simulated dependency of the 3D point standard deviation *σ*_3D_ and point cloud completeness *p* on the degree of projector defocusing, which is approximated by a Gaussian blur with standard deviation *σ*_blur_, and the rotational speed *ω* of the GOBO wheel. **d** A detailed view of the GOBO wheel of the sensor that is shown in **a**. Undesired opaque spots, which may negatively affect the measurement accuracy, are clearly recognisable

It is useful to experimentally verify the simulation results. However, the diagram that is shown in Fig. 2a suggests that it is not practical to experimentally vary all five parameters that have been considered so far: *c*, *σ*_blur_, *n*, *ω*, and *e*. Manufacturing many GOBO wheels to vary the number of fringes *n* and the fringe width ratio *c* would be very cumbersome and expensive. Therefore, we decided to use one of our existing sensors (see Fig. 3a)^46,47^ and varied the projection blur (in the form of the standard deviation *σ*_blur_ of a Gaussian blur) and the rotational speed *ω* = *ϕ*/*T* of the GOBO wheel.

For each combination of *σ*_blur_ and *ω*, we recorded 1000 images of the aperiodic sinusoidal fringes that were projected onto a granite slab. We used 10 images to calculate each 3D point cloud, thereby obtaining 100 independent data sets per parameter combination. Figure 3b, c shows the resulting average 3D standard deviation *σ*_3D_ and average point cloud completeness *p*, respectively, as functions of *σ*_blur_ and *ω*. The lowest achievable degree of defocus leads to the highest accuracy. Moreover, the standard deviation and completeness show an opposite trend within the scanned parameter range: whereas for a high measurement accuracy, the rotational speed should be comparatively low, the completeness of the calculated point clouds increases with increasing rotational speed. A suitable parameter combination is, e.g., *σ*_blur_ = 15 µm and *ω* = 0.15°/*T*, which results in a 3D standard deviation of 150 µm and a completeness of 100%.

To determine whether the experimental measurement data match the theoretically expected results, we simulated the 3D data generation of the NIR scanner in the same way as described in the Methods section, but with modified parameters that took the deviating sensor geometry into account (see Table 3). The first step (the random generation of the GOBO wheel) was obsolete, as the design of the wheel was known. For the dark and shot noise that we added to the rendered camera images, we assumed values that corresponded to a signal-to-noise ratio of SNR ≈ 17 dB. In the simulation, we were able to vary the two parameters, namely, *σ*_blur_ and *ω*, more finely and within a larger range of values than in the experiment. The resulting diagrams for the standard deviation and completeness are shown in Fig. 3e, f, respectively. The optimum results are achieved for *σ*_blur_ = 7.5 µm and *ω* = 0.17°/*T*. For this parameter combination, the 3D standard deviation is *σ*_3D_ ≈ 130 µm and the completeness is *p* = 100%.

**Table 3:** Parameters of the GOBO projection-based NIR 3D sensor that was used to experimentally verify the theoretical results

Parameter	Value	Parameter	Value
*N*	10	*d*	0.5 m
*n*_tot_	946	$v = \frac{s}{d}$	1
$\phi _{{\rm{avg}}} = \frac{{360^\circ }}{{n_{{\rm{tot}}}}}$	0.38°	$M = \frac{s}{a}$	43
$c = \frac{{\phi _{{\rm{max}}}}}{{\phi _{{\rm{min}}}}}$	2.5	*w*	1.5 m
*P*(*φ*)	Uniform dist.	$\omega = \frac{\varphi }{T}$	Varied
*r*	23.9 mm	$e = \frac{{t_{{\rm{exp}}}}}{T}$	0.95
*a*	11.6 mm	*l*	0.23 m
*δ*	35.5°	*γ*	8.8°
*n*	94	*α*	18.2°
*σ*_blur_	Varied	*A*	1 Mpx
*s*	0.5 m	*b*	8 bit

Even if the simulated values very well agree with the experimental results, it is likely that the comparatively high assumed camera noise (SNR ≈ 17 dB) is lower in practice and the 3D accuracy is negatively affected by another effect. There are many indications that the GOBO wheel itself plays a major role since its production quality is not optimal. According to Fig. 3d, especially in the magnified view, the fringes on the GOBO wheel are strongly frayed and there are many dark spots in areas that should be transparent. The GOBO wheel that is shown here and was used in the NIR scanner was manufactured by applying an aluminium layer onto a 1.1 mm thick substrate of borosilicate glass, which was partially removed by a laser beam. Due to the recognisable artefacts, we will evaluate alternative fabrication methods. In the near future, we are planning to produce GOBO wheels via electron-beam lithography.

## Discussion

We have studied the performance of the novel principle of 3D shape measurement using GOBO-projected aperiodic sinusoidal patterns^25^. For this purpose, we have varied five key parameters that influence the quality of the measurement result (e.g., the number of fringes and the rotational speed of the GOBO wheel) in an extensive rendering simulation of the measurement of a plane object. Two main conclusions are drawn from the results of our investigations. First, when setting up a GOBO projection-based 3D sensor, the parameters must be tuned carefully, as small variations of parameters can lead to substantially different 3D results. Second, it is possible to identify parameters of a GOBO-projected pattern that ensure a small standard deviation and high completeness of the 3D point cloud. In this way, e.g., 10 GOBO-projected aperiodic sinusoidal patterns can lead to the same measurement accuracy as 5 to 6 GOBO-projected phase-shifted sine-like patterns, but without requiring phase unwrapping. This result confirms the findings of previous investigations of the principle of projecting aperiodic sinusoidal patterns^26,27^.

**Fig. 4:**
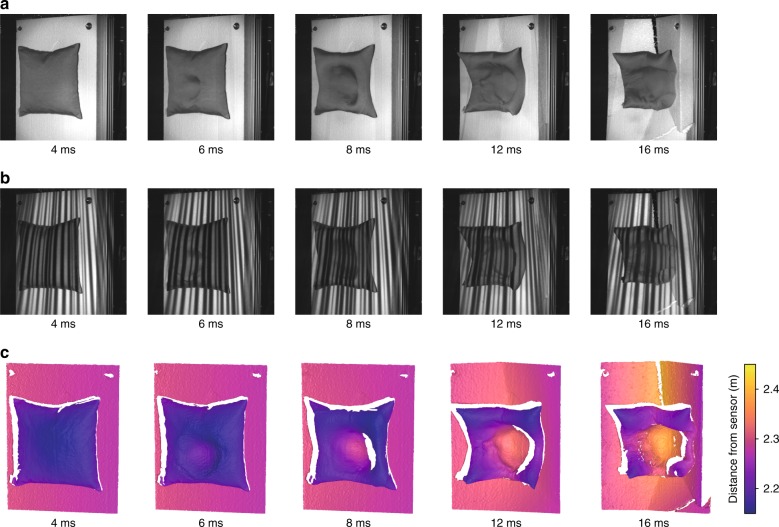
**High-speed 3D measurement of the impact of a 40-bar nitrogen jet on a 400** **×** **400** **mm**^**2**^ **pillow that is attached to a polystyrene plate.** **a** Snapshots of the recorded process at five points in time. **b** Camera images of the GOBO-projected aperiodic sinusoidal fringes, which are recorded with a resolution of 512 × 408 px at a frame rate of 55.2 kHz. **c** Reconstructed point clouds at a 3D rate of 5.5 kHz (see also Supplementary Video S2)

Since corresponding points are detected solely via temporal correlation, the results that are obtained for the plane measurement object can be generalised to arbitrary objects (see Supplementary Video S1), demonstrating the excellent suitability of GOBO projection of aperiodic sinusoidal patterns for high-speed 3D shape measurement, which in addition to the generation of point clouds with high accuracy and completeness, is characterised by the potential for very fast pattern variation and high radiant flux. The results of the exemplary measurement that is shown in Fig. 4 demonstrate this, as we were able realise a 3D rate of 5.5 kHz, which corresponds to a temporal resolution of just over 180 µs (see also Supplementary Video S2). Moreover, due to the pattern generation via a metal mask, a GOBO projector is suitable for application in a wide spectral range that is beyond the visible light range.

Because of its benefits, the principle of GOBO projection should be further investigated. Future research should focus on studying the dependence of the 3D measurement quality on the number of projected patterns and on the motion of the measurement object. The projection of a smaller number of patterns generally results in lower measurement accuracy, point cloud completeness, and/or density. However, it might be reasonable to use fewer patterns for 3D reconstruction of fast moving objects to minimise the acquisition time. Therefore, it is of particular interest to examine up to which object speeds the GOBO projection of a series of aperiodic sinusoidal patterns is superior to common single-shot methods.

## Materials and methods

### Simulation framework

To identify an optimal set of GOBO wheel parameters within a simulation framework, not all of the parameters that are shown in Table 1 should be freely varied because the solution space would be inconveniently large and some of the parameters are not independent. For instance, equally scaling the GOBO wheel and the size of the illuminated area by a specified factor will lead to the same results, as the cameras observe the same patterns. Therefore, we simulate a typical sensor setup and set some of the parameters, e.g., a distance of 200 mm between the cameras, a working distance of 1 m, and a measurement volume of 300 × 300 × 300 mm^3^ (see the last column of Table 1). Hence, the optimisation problem is reduced to a five-dimensional problem with the following variables:

- *c* = 1…10 (the ratio of the maximum and minimum strip or slit widths),
- *σ*_blur_ = 0…50 µm (the degree of defocusing of the GOBO wheel),
- *n* = 20…400 (the number of illuminated strips + the number of illuminated slits),
- *ω* = 0.001°/*T*…2.5°/*T* (the rotational speed of the GOBO wheel), and
- *e* = 0.5…1 (the ratio of the camera exposure time *t*_exp_ and period *T*).

**Fig. 5:**
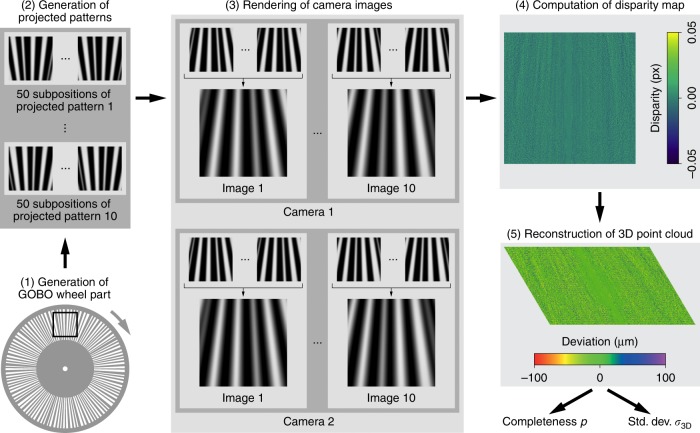
Block diagram of the simulation framework. According to the parameters *c* and *n*, a part of a GOBO wheel is generated (1). Based on the parameters *σ*_blur_, *ω*, and *e*, subpatterns of the rotating GOBO wheel are determined (2). The subpatterns that are related to a pattern are projected onto a plane and the corresponding camera images are rendered (3). After computing the disparity map (4) and reconstructing the 3D point cloud (5), the standard deviation *σ*_3D_ and completeness *p* are estimated

Figure 5 shows a block diagram of the simulation framework. The procedure can be divided into the five steps explained in next sections.

#### Generation of the GOBO wheel

First, *n* random values *X*_*i*_ are generated, each between 1 and *c*. Then, the values

3 $$\phi _j = \delta \frac{{\mathop {\sum }\nolimits_{i = 1}^j X_i}}{{\mathop {\sum }\nolimits_{i = 1}^n X_i}}$$

represent the angles of change between transparent and opaque fringes in the illuminated part of the GOBO wheel (0 < *φ*_*j*_ ≤ *δ*). The resulting GOBO mask contains *n* strips and slits, which each cover an angle between *φ*_min_ and *φ*_max_ = *cφ*_min_ ≥ *φ*_min_.

#### Generation of the projected patterns

The GOBO wheel is continuously rotating at a speed *ω* = *ϕ*/*T*, i.e., between successive image acquisitions, the GOBO wheel has been rotated by an angle of *ϕ*. However, to take the movement during the exposure time and the ratio of the exposure time *t*_exp_ and period *T* into account, the wheel is rotated by subangles *ϕ*_*i*_. We opted for 50 subpatterns: *ϕ*_*i*_ = *iϕe*/50. For each of the 50 subpositions, a square of size *a* × *a* = 10 × 10 mm^2^ with a distance of *r* = 25 mm from the GOBO wheel centre is cut out. The defocusing of the imaging lens is approximated by a Gaussian blur with standard deviation *σ*_blur_.

#### Rendering of the camera images

The generated GOBO subpatterns are projected onto a plane that is parallel to the GOBO wheel at a working distance of 1 m. The corresponding camera images with a resolution of 1024 × 1024 px are rendered using a ray-tracer on the basis of a physically based rendering system, namely, “PBRT”^48^. Each set of 50 subimages is averaged to form the camera image, thus keeping motion blur in mind. Altogether, *N* = 10 images per camera are generated, which are rectified according to the camera-projector-camera arrangement^21,49^. In the rectified images with coordinates $\left( x{\prime}_{\hskip-3pt1} ,y\prime_{\hskip-3pt1} \right)$ and $\left( {x\prime_{\hskip-3pt2} ,y\prime_{\hskip-3pt2} } \right)$, corresponding points lie on the same horizontal line, i.e., $y\prime_{\hskip-3pt1} = y\prime_{\hskip-3pt2}$.

#### Computation of the disparity map

The rectified images are used to calculate the coefficient *ρ* of the normalised cross-correlation according to Eq. 1 between each pixel in camera 1 and pixels on the same horizontal line in camera 2. The search area is limited by the measurement volume, as the distance of *w* ± *d*/2 = (1 ± 0.15) m from the sensor corresponds to a disparity search range of ≈ ± 110 px. The global maximum of the correlation coefficient within this disparity search range is considered to occur at the corresponding point. Subpixel accuracy is achieved via linear grey value interpolation between adjacent pixels in each rectified image of camera 2, with the aim of maximising *ρ*.

#### Reconstruction of the 3D point cloud

Based on the disparity map, for each pair of corresponding points, namely, $\left( {x\prime_{\hskip-3pt1} ,y\prime } \right)$ and $\left( {x\prime_{\hskip-3pt2} ,y\prime } \right) = \left( {x\prime_{\hskip-3pt1} - {{disp}},y\prime } \right)$, a point with homogeneous coordinates

4 $${\mathbf{Q}}\left( {\begin{array}{*{20}{c}} {x\prime_{\hskip-3pt1} } \\ {y\prime } \\ {{\rm{disp}}} \\ 1 \end{array}} \right)\quad {\rm{with}}\,{\mathbf{Q}} = \left[ {\begin{array}{*{20}{c}} 1 & 0 & 0 & { - c\prime_{\hskip-3ptx1} } \\ 0 & 1 & 0 & { - c\prime_{\hskip-3pty1} } \\ 0 & 0 & 0 & \kappa \\ 0 & 0 & { - 1/l} & {\left( {c\prime_{\hskip-3ptx1} - c\prime_{\hskip-3ptx2} } \right)/l} \end{array}} \right]$$

can be calculated, where *κ* is the camera constant of the rectified system (in pixel units), $c\prime_{\hskip-3ptx1}$ and $c\prime_{\hskip-3pty1}$ are the coordinates of the (rectified) principal point in camera 1, and $c\prime_{\hskip-3ptx2}$ is the *x*-coordinate of the (rectified) principal point in camera 2. The resulting point cloud is compared with the known plane so that outliers can be identified. The point cloud completeness *p* is given by the ratio of the number of correct points *m*_correct_ and the maximum possible number of points *m*_max_:

5 $$p = \frac{{m_{{\rm{correct}}}}}{{m_{{\rm{max}}}}} = 1 - \frac{{m_{{\rm{false}}}}}{{m_{{\rm{max}}}}}$$

After removing the *m*_false_ outliers, the standard deviation *σ*_3D_ of the remaining 3D points from the plane is calculated.

To take the various types of noise of real cameras into account, step 3 (i.e., Rendering of the camera images) can be extended by following EMVA standard 1288^28^. Let the output of the ray-tracer be the number of photons *μ*_*p*_ that impinge on each camera pixel. Depending on the total quantum efficiency *η*(*λ*), they generate a number of electrons, which is expressed as *μ*_*e*_ = *η*(*λ*)*μ*_*p*_. Without loss of generality, we set *η*(*λ*) = 1. Then, the number of electrons *μ*_*e*_ = *μ*_*p*_ fluctuates with a signal-dependent shot noise *n*_*e*_ (Poisson distribution with standard deviation $\sigma _e = \sqrt {\mu _e}$) and a signal-independent dark noise *n*_*d*_ (normal distribution with standard deviation *σ*_*d*_). The noisy number of electrons

6 $$\mu _e^{{\rm{noisy}}} = n_e + n_d$$

is converted to a grey value $g^{{\rm{noisy}}} = K\mu _e^{{\rm{noisy}}}$ according to the overall system gain *K*. After clipping the grey value to the dynamic range of the camera, namely, 0…2^*b*−1^, it is rounded to the nearest integer to take analogue-to-digital conversion into account. The signal-to-noise ratio (SNR) can be expressed as

7 $${\rm{SNR}} = \frac{{\mu _e}}{{\sqrt {\sigma _d^2 + \sigma _q^2/K^2 + \mu _e} }}$$

with the variance $\sigma _q^2 = 1/12\,{\rm{DN}}$ of the uniformly distributed quantisation noise. In addition to the absence of shot and dark noise, we have decided to simulate three reasonable levels of noise, which are described as follows:

- *K* = 1/25 DN/*e*^−^, *σ*_*d*_ = 0.5/*K* (low noise),
- *K* = 1/10 DN/*e*^−^, *σ*_*d*_ = 1/*K* (medium noise), and
- *K* = 1/5 DN/*e*^−^, *σ*_*d*_ = 2/*K* (high noise).

For a grey value of *g* = 255, these levels correspond to signal-to-noise ratios of SNR ≈ 19 dB, 17 dB, and 15 dB. When only considering quantisation noise (i.e., *K*→∞ and *σ*_*d*_→0), the signal-to-noise ratio is SNR ≈ 29 dB.

In this way, we generated several thousand random parameter combinations and evaluated the resulting point clouds with respect to the standard deviation *σ*_3D_ and completeness *p*. Then, we compared the results with those that were obtained from GOBO-projected phase-shifted sine-like patterns, as phase-shifting fringe projection is considered the gold standard for structured light-based 3D measurement. The simulation framework can easily be used to carry this out by setting *c* = 1 (the ratio of the maximum and minimum fringe widths) and *ω* = 2*φ*_avg_/*NT* (the rotational speed of the GOBO wheel).

### Experimental setup

To experimentally verify the simulation results, we used one of our existing GOBO projection-based sensors (see Fig. 3a)^46,47^. Since this 3D scanner is primarily intended for the irritation-free measurement of human faces, the aperiodic sinusoidal patterns are projected and detected in the near infrared (NIR) region at a wavelength of 850 nm. With the help of an additional RGB camera, colour information can be acquired simultaneously with the 3D measurement.

For our measurements, we used two Basler “acA2040-180kmNIR” measurement cameras at a resolution of 1024 × 1024 px and a frame rate of *f* = *T*^−1^ = 50 Hz. By using an exposure time of *t*_exp_ = 19 ms, we set the exposure time ratio to *e* = *t*_exp_/*T* = 0.95. The cameras’ projection centres were of distance *l* = 0.23 m from each other and they observed a measurement field of size *s *× *s* = 0.5 × 0.5 m^2^ at a working distance of *w* = 1.5 m. The GOBO projector contained a GOBO wheel with a diameter of 66 mm. The GOBO wheel comprised *n*_tot_ = 946 strips and slits of various widths with a known distribution, ~94 of which were illuminated at any point in time. All relevant parameters are summarised in Table 3. The grey highlighted cells indicate the two parameters that we have varied in the experiment: the projection blur (in the form of the standard deviation *σ*_blur_ of a Gaussian blur) and the rotational speed *ω* = *ϕ*/*T* of the GOBO wheel.

To determine the standard deviation *σ*_blur_ that corresponds to a specified level of projector defocusing, we placed a matte white sprayed planar granite slab with a calibrated peak-to-valley height of 4.55 µm parallel to the sensor at the working distance and illuminated it with the pattern of the stationary GOBO wheel. The resulting camera image was compared with differently blurred camera images of (hypothetical) binary fringes. The value *σ*_blur_ for which the sum of the squared deviations was minimal was assigned to the respective defocusing setting. Due to the inherent slight lens blur, a value of *σ*_blur_ = 0 could not be realised. In addition, excessively high values could not be achieved because defocusing settings that exceed a specified level produce additional effects that cannot be approximated by a Gaussian blur. Overall, we have realised values between *σ*_blur_ ≈ 15 µm and 60 µm. The rotational speed of the GOBO wheel was varied such that the wheel was rotated between 0.05° and 0.50° between two consecutive acquisition trigger signals.

## Electronic supplementary material

Supplementary Movie 1

Supplementary Movie 2

Supp information
