# Supplementary material for: GOBO projection for 3D measurements at highest frame rates: a performance analysis
Source: Light Sci Appl. 2018 Oct 3;7:71. doi: 10.1038/s41377-018-0072-3 (PMC6168569; doi:10.1038/s41377-018-0072-3)
Supplement: Supplementary file 3 — Supp information [file 41377_2018_72_MOESM3_ESM.docx]

Supplementary Information for

GOBO projection for 3D measurements at highest frame rates: A performance analysis

**Stefan Heist^1,2,*^, Patrick Dietrich^1,2^, Martin Landmann^1,2^, Peter Kühmstedt^2^, Gunther Notni^2,3^ & Andreas Tünnermann^1,2^**

^1^ Institute of Applied Physics, Abbe Centre of Photonics, Friedrich Schiller University, 07745 Jena, Germany

^2^ Fraunhofer Institute for Applied Optics and Precision Engineering IOF, 07745 Jena, Germany

^3^ Department of Mechanical Engineering, Ilmenau University of Technology, 98693 Ilmenau, Germany

^*^ Correspondence: Stefan Heist, Institute of Applied Physics, Abbe Centre of Photonics, Friedrich Schiller University, Albert-Einstein-Str. 15, 07745 Jena, Germany, stefan.heist@uni-jena.de, +49 3641 807-214 (phone), +49 3641 807-602 (fax)

[stefan.heist@uni-jena.de](mailto:stefan.heist@uni-jena.de), [patrick.dietrich@uni-jena.de](mailto:patrick.dietrich@uni-jena.de), [martin.landmann@uni-jena.de](mailto:martin.landmann@uni-jena.de), [peter.kuehmstedt@iof.fraunhofer.de](mailto:peter.kuehmstedt@iof.fraunhofer.de), [gunther.notni@tu-ilmenau.de](mailto:gunther.notni@tu-ilmenau.de), [andreas.tuennermann@iof.fraunhofer.de](mailto:andreas.tuennermann@iof.fraunhofer.de)

# Supplementary videos


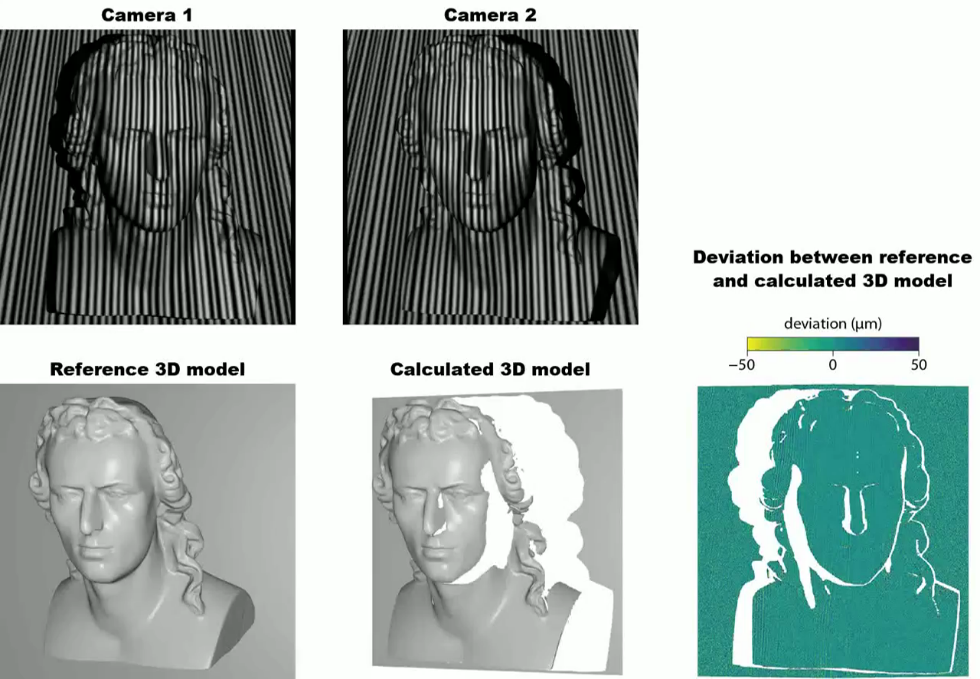


Supplementary Video S1 Simulated 3D measurement of a bust of the German poet and philosopher Friedrich Schiller by using optimized GOBO-projected aperiodic sinusoidal fringes.


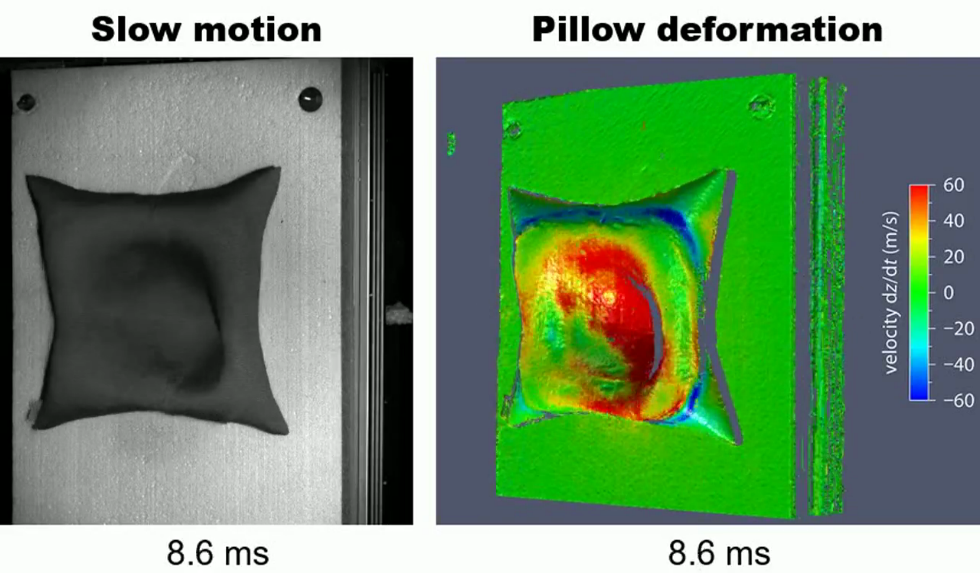


Supplementary Video S2 High-speed 3D measurement of the impact of a 40-bar nitrogen jet on a 400 × 400 mm^2^ pillow attached to a polystyrene plate, recorded at a 3D rate of 5.5 kHz.
